# Recent developments of protein kinase inhibitors as potential AD therapeutics

**DOI:** 10.3389/fncel.2013.00189

**Published:** 2013-11-19

**Authors:** Volkmar Tell, Andreas Hilgeroth

**Affiliations:** Research Group of Drug Development and Analysis, Institute of Pharmacy, Martin Luther University Halle-WittenbergHalle, Germany

**Keywords:** protein kinase, inhibitor, tau phosphorylation, small molecules, structure–activity relationships, inhibitor binding

## Abstract

Present Alzheimer’s disease (AD) therapies suffer from inefficient effects on AD symptoms like memory or cognition, especially in later states of the disease. Used acteylcholine esterase inhibitors or the NMDA receptor antagonist memantine address one target structure which is involved in a complex, multifactorial disease progression. So the benefit for patients is presently poor. A more close insight in the AD progression identified more suggested target structures for drug development. Strategies of AD drug development concentrate on novel target structures combined with the established ones dedicated for combined therapy regimes, preferably by the use of one drug which may address two target structures. Protein kinases have been identified as promising target structures because they are involved in AD progression pathways like pathophysiological tau protein phosphorylations and amyloid β toxicity. The review article will shortly view early inhibitors of single protein kinases like glycogen synthase kinase (gsk3) β and cyclin dependent kinase 5. Novel inhibitors will be discussed which address novel AD relevant protein kinases like dual-specificity tyrosine phosphorylation regulated kinase 1A (DYRK1A). Moreover, multitargeting inhibitors will be presented which target several protein kinases and those which are suspected in influencing other AD relevant processes. Such a multitargeting is the most promising strategy to effectively hamper the multifactorial disease progression and thus gives perspective hopes for a future better patient benefit.

## INTRODUCTION

Presently Alzheimer’s disease (AD) therapies are limited by the availability of just two groups of drugs with one group consisting of just one drug. Acetylcholine esterase inhibitors increase the neuronal acetylcholine amounts, whereas memantine, an antagonist at the neuronal *N*-methyl D-aspartate (NMDA) receptor, reduces a neuronal overstimulation caused by the neurotransmitter glutaminic acid (Birks, 2006; McShane et al., 2006). Both the loss of acetylcholine and the neuronal overstimulation contribute to the decay of neurons and thus the AD progression. However, the patient benefit of the present drugs is poor and limited to the early stage of the disease (Terry and Buccafusco, 2003; Raina et al., 2008). Memory, cognition, and daily behavior for life managing is hardly improved by the drugs in later stages of a severe AD (Terry and Buccafusco, 2003; Raina et al., 2008). It is known that AD is a multifactorial disease which means that different pathophysiological factors all contribute to the AD progression (Iqbal and Grundke-Iqbal, 2002). Most important hallmarks are the protein deposites which are found inside and outside the neuronal cells, namely the extracellular Aβ plaques and the intracellular neurofibrillary tangles (NFTs; Mohandas et al., 2009; Piau et al., 2011).

However, both proteins and their precursors are known to play a central role in the neuronal decay and disease progression. While a toxicity of the Aβ plaques is still under debate, their soluble precursors of non-aggregated Aβ proteins are toxic in various ways also by the formation of NFTs as a result of aggregation of a hyperphosporylated and misfolded tau protein as will be discussed later (Mattson, 2004; Pakaski and Kalman, 2008; Mohandas et al., 2009; Piau et al., 2011). Such tau protein can no longer support the intracellular transport mediated by the microtubules and with the loss of neuronal function the cell is dedicated to undergo apoptosis (Iqbal and Grundke-Iqbal, 2008; Marco et al., 2010). Protein kinases are known to mainly contribute to these toxic events as they play a central role in the cellular pathways of regulated cell function and division.

With the understanding of various protein kinase functions the question of developing inhibitors as potential AD therapeutics has arisen. Protein kinase inhibitors are long established in cancer therapies where they regulate the overactivity of protein kinases which lead to uncontrolled cell divisions, cell migration, and cellular invasion (Krug and Hilgeroth, 2008; Zhang et al., 2009). The toxicity of such anticancer protein kinase inhibitors has always been a critical question of causing toxic or undesired side-effects (Liao and Andrews, 2007). Viewing the years of research in this field multitargeting protein kinase inhibitors established and are well tolerated by patients with only limited side-effects (Krug and Hilgeroth, 2008). So there are certain perspectives that protein kinase inhibitors for AD therapy may show promising effects in the pathophysiological AD process on one hand and for a more effective therapy on the other hand with respect to the knowledge that present drugs are no real perspective drugs to effectively influence the disease progression. The review will give a short summary of the early protein kinase inhibitors which target early known single target structures. Such early protein kinase inhibitors have been developed to reduce the activity of tau protein hyperphosphorylating kinases which have been found partly overactive or overexpressed in respective neuronal cells in AD brains and thus mainly contribute to the aggregation and loss of function of the hyperphosphorylated tau. Then novel inhibitors will be viewed which address novel targets with evidence for the AD progression and those which are dedicated to a multitargeting of more than one target structure.

## EARLY PROTEIN KINASE INHIBITORS

Tau protein has been the main target structure for protein kinase inhibitors since tau protein was found hyperphosphorylated in AD brains. The tau protein hyperphosphorylation results in dissociation from the microtubules the function of which is supported by tau protein (Martin et al., 2013). Moreover, the hyperphosphorylation causes a loss of solubility and leads to the formation of paired helical filaments (PHFs) which further aggregate to NFTs (Avila, 2006; Martin et al., 2011, 2013). The reason for the tau protein hyperphosphorylation is an imbalance of phosphorylation and dephosphorylation of tau. This imbalance is partly driven by a reduced tau protein dephosphorylation or by an overactivity of the phosphorylating protein kinases (Iqbal and Grundke-Iqbal, 2002; Tian and Wang, 2002; Pei et al., 2008; Martin et al., 2013). Glycogen synthase kinase (GSK) 3β plays a central role in the tau phosphorylation process. It has been reported that 31% of the pathological phosphorylation sites of tau protein are phosphorylated by GSK3β (Martin et al., 2013). GSK3β has been found co-localized with NFTs and is found overexpressed in AD brains with increased activity (Pei et al., 1997; Leroy et al., 2007). Toxic Aβ is known to increase the activity of GSK3β which contributes to an increased Aβ production via the tau phosphorylation (Hoshi et al., 1996; Takashima et al., 1996). Therefore GSK3β forms a link of Aβ toxicity and tau pathology (Martin et al., 2013). Lithium as an early GSK3β inhibitor reduced tau phosphorylation and prevented reversed aspects of tau pathology in animal models (Tariot and Aisen, 2009). However, treatment of AD patients with mild AD in the early disease states showed no improvement in cognition (Hampel et al., 2009). The result correlated with unchanged AD biomarkers in the cerebrospinal fluid (CSF) of the patients, namely phosphorylated tau, total tau, and toxic Aβ (Hampel et al., 2009). Several GSK3β inhibitors are under development belonging to the paullone, indirubin, and the maleimide families. However, no representative of these inhibitor groups reached clinical trials so far. Reasons for their failure have been proven cytotoxic effects.

The other important tau protein kinase which is involved in pathophysiological tau protein phosphorylation is the cyclin dependent kinase 5 (cdk5). The normally cdk5 regulating protein p35 is found truncated in AD brains to 25 amino acids. This protein p25 leads to a constitutive activation of cdk5 and thus causes the pathophysiological tau phosphorylation (Patrick et al., 1999). The hyperphosphorylated tau protein dissociates from the microtubules and forms NFTs on cdk5 induction by p25 (Cruz et al., 2003; Noble et al., 2003). Also NFTs are phosphorylated by cdk5 action (Baumann et al., 1993; Hollander et al., 1996). Cdk5 phosphorylated tau becomes a better substrate for GSK3β so that excessive tau phosphorylation proceeds (Sengupta et al., 1998). Moreover, cdk5 promotes apoptosis in AD brains which may follow Aβ toxicity influenced by the hyperphosphorylated tau or by remaining p35 protein (Hamdane et al., 2005; Utreras et al., 2009).

From the number of cdk inhibitors which were all non-selective like flavoperidol a pan-cdk inhibitor investigated for cancer therapy, cdk5-selective so-called CP-inhibitors were reported with nanomolar affinities and the ability to cross the blood brain barrier (Wen et al., 2008). They were described to reduce increased Aβ levels on p25 overexpression. However, as far as known these inhibitors remained in preclinical states.

## NOVEL INHIBITORS

### SINGLE PROTEIN KINASE TARGETING OF CDK5, P38, JNK, Erk, CK, and DYRK1A

**FIGURE 1:**
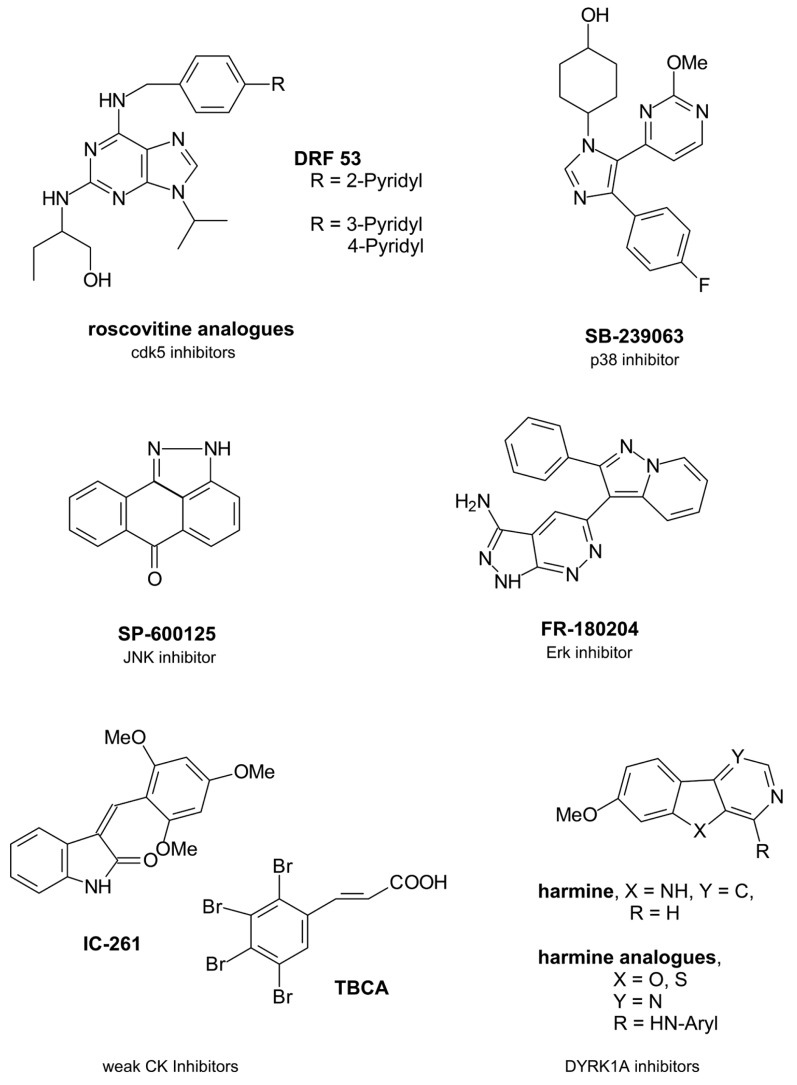
**Single protein kinase-targeting inhibitors**.

Due to the significant role of cdk5 in tau phosphorylation there have been ongoing efforts to develop novel inhibitors of the kinase which have been structurally based on the cdk inhibitor roscovitine (seliciclib) as a trisubstituted purine compound (**Figure 1**).

Beside cdk5 roscovitine inhibits several cdks like cdk1, cdk2, or cdk9 in micromolar concentrations (Bettayeb et al., 2008). It presently undergoes clinical trials of various types of cancer (U.S. National Institutes of Health, 2010). However, roscovitine crosses the blood brain barrier and this advantage might have driven further studies because only about 2% of small molecule inhibitors are able to penetrate into the CNS (Meijer and Raymond, 2003; Pardridge, 2005). One structurally varied compound has been DRF 53 with a 2-pyridyl residue attached to the 4-position of the benzylamine substituent. With a similar cdk inhibition profile the toxic properties increased compared to roscovitine so that it is suggested to be used as anticancer agent like roscovitine (Bettayeb et al., 2008). In a recent paper the 2-pyridyl residue has been changed by both a 3- and a 4-pyridyl residue (Demange et al., 2013). More favourable cdk5 inhibition data resulted in nanomolar ranges. Docking studies in the ATP binding pocket demonstrated a hydrogen bonding of the 4-pyridyl nitrogen to the Nε of Lys89 of the protein backbone to explain the increased activity. However, beside cdk5 also cdk2 was inhibited and all derivatives were toxic (Demange et al., 2013).

P38 protein kinase belonging to the mitogen activated protein kinases (MAPKs) is a tau protein phosphorylating kinase which contributes to tau protein dissociation from the microtubules and facilitates a further tau aggregation (Martin et al., 2013). In AD brains Aβ activates p38 isoforms p38α und p38β via activated glia (Rojo et al., 2008). In the following proinflammatory cytokines are produced like interleukin-1β (IL-1β) and tumor necrosis factor α (TNFα) (Shaftel et al., 2008). So nanomolar active p38 inhibitor SB-239063 is able to reduce the inflammatory cytokine production (Underwood et al., 2000).

Erk isoforms 1 and 2 contribute to abnormal tau protein phosphorylation including the formation of pathological tau conformations (Pei et al., 2002; Martin et al., 2013). So the inhibition of Erk promises a reduced tau pathology. FR-180204 is the sole selective Erk inhibitor described so far (Ohori et al., 2005).

Finally, JNKs of the MAPK family have been interesting target structures because they also contribute to tau phosphorylation. JNK activation is mediated by toxic Aβ fragments (Philpott and Facci, 2008). However, activated JNKs also increase the γ-secretase activity and thus contribute to increased toxic Aβ levels resulting from the amyloid precursor protein (APP) by γ-secretase cleavage (Shen et al., 2008). Both MAPKs p38 and JNK have been co-purified with NFTs in AD brains (Zhu et al., 2000). So JNKs are potential AD-therapeutic target structures. SP-600125 is a potent JNK isoform inhibitor (Bennett et al., 2001). However, JNK3 is the known isoform which mediates the toxic response to Aβ with a greater role in the regulation of toxicity in brain than the other isoforms JNK1/2 (Philpott and Facci, 2008). So it would be of benefit to develop a more exclusive JNK3 inhibitor.

Casein kinase isoforms CK1δ and CK1ε are expressed in brain and are mainly involved in the pathophysiological tau phosphorylation with 26% of those tau amino acids being phophorylatated by CK1 (Knippschild et al., 2005; Martin et al., 2013). CK1δ mRNA levels as well as the kinase itself are found mainly increased in AD brains up to 30-fold (Yasojima et al., 2000). CK1 has been found co-localized with NFTs like GSK3β being active in the tau phosphorylation of NFTs (Kuret et al., 1997; Schwab et al., 2000). Moreover, CK phosphorylation of tau sites facilitates the dissociation from the microtubules (Martin et al., 2013). Similar to GSK3β Aβ peptides activate CK thus triggering the tau pathology which on the other hand increases Aβ (Takashima et al., 1996; Flajolet et al., 2007). Furthermore, CK1ε is known to play a role in the APP processing, likely by regulating the activity of γ-secretase which contributes to the formation of the toxic Aβ peptides. CK is additionally a priming kinase for GSK3β and has a regulatory role in cdk5 function (Knippschild et al., 2005).

Thus, CK is an interesting target structure for AD relevant protein kinase inhibitors, because the AD overactivity of the kinase may be regulated by an inhibitor without effecting the basic activity in cells. The presently known CK inhibitors IC-261 and tetrabromocinnamic acid (TBCA) are poor inhibitors with activities in partly higher micromolar ranges (Mashhoon et al., 2000; Pagano et al., 2007).

Dual-specificity tyrosine phosphorylation-regulating kinase 1A (DYRK1A) phosphorylates tau and transcription factor cAMP Response Element Binding (CREB) which is involved in learning and memory (Yang et al., 2001). Aβ peptides increase DYRK1A mRNA levels in AD brains (Kimura et al., 2007). However, tau phosphorylation of DYRK1A is reported to be triggered by GSK3β (Kimura et al., 2007). Interestingly, DYRK1A phosphorylates serine 202 of tau protein and this phosphorylation induces a conformational change of tau which is pathological (Martin et al., 2013). Thus, DYRK1A emerged to an interesting protein kinase for a tau-directed therapy. Harmine is an early DYRK1A inhibitor which inhibits not only the enzyme itself but also its tyrosine autophosphorylation (Seifert et al., 2008). While harmine is a promising inhibitor with activities in nanomolar ranges, recent structural changes in the molecular skeleton by replacing the indole nitrogen by an oxygen or a sulfur atom and by replacing the annelated pyridine in the β-carboline scaffold by a pyrimidine led to decreases in activity (Loidreau et al., 2013). Moreover, it remains of doubt whether DYRK1A is a favourable target kinase because an inhibition would have negative consequences for the CREB phosphorylation. This phosphorylation is necessary for learning and memory processes which are both impaired in AD patients.

### MULTITARGETING

**FIGURE 2:**
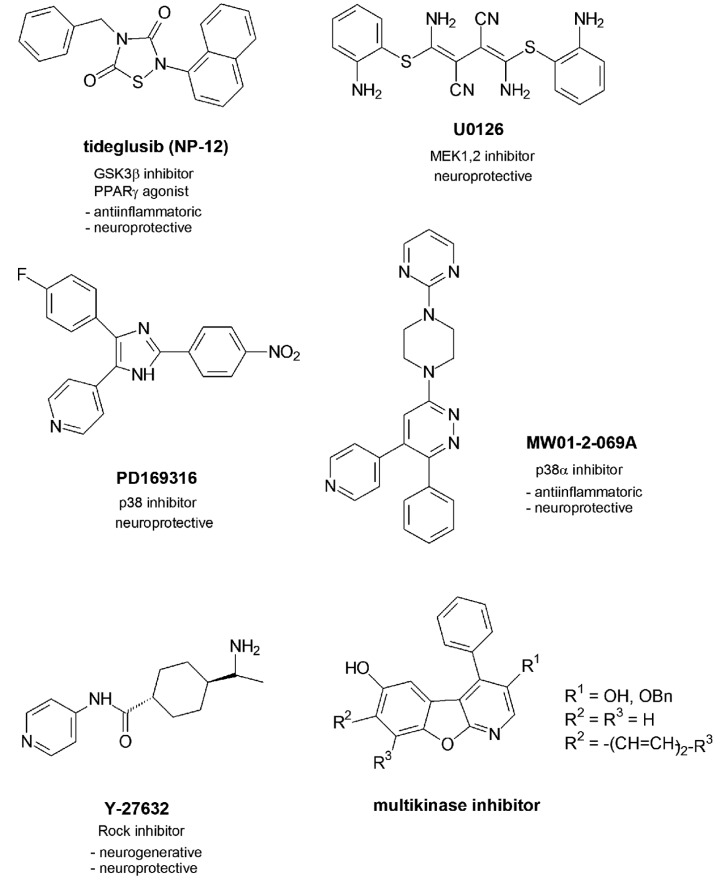
**Multitargeting inhibitors**.

One GSK3β inhibitor named tideglusib (NP-12) with a thiadiazolidinone scaffold presently undergoes phase 2 of clinical trials (**Figure 2**).

In contrast to the other GSK3β inhibitors which are competitive inhibitors of ATP in the ATP-binding pocket NP-12 is a non-competitive inhibitor which was reported to reduce tau phosphorylation and amyloid depositions in brain and prevent neuronal death and cognitive deficits in animal models (http://clinicaltrials.gov/ct2/results?/term = Noscira, 2010). Moreover, NP-12 was identified as nuclear receptor PPARγ agonist which mediated effective anti-inflammatory and neuroprotective properties (Luna-Medina et al., 2007).

In recent studies two inhibitors of the MAPK family have been investigated in their effects in Aβ-injected rats (Ashabi et al., 2012). U0126 is an inhibitor of MEK1 and 2 which regulates the activity of Erk1 and 2, while PD169316 is a p38 inhibitor. Aβ activates MAPKs p38, Erk, and JNK. This activation causes a mitochondrial dysfunction by disturbance of the mitochondrial biogenesis. In early states of AD this mitochondrial abnormality is responsible for brain energy depletions. Aβ causes deficits in learning, memory and cognition as demonstrated by the behavioral changes of the rats after Aβ-treatment. The inhibitor applications led to increased levels of c-fos as activated gene which contributes to long-term memory processes. Moreover, the levels of CREB increased. The transcription factor is not only involved in early processes of long-term memory as discussed but also regulates the expression of factors PGC-1α and of NRF-1 (Kudo et al., 2005; Vercauteren et al., 2006). Both factors play a role in the biogenesis of mitochondria which are dysregulated in pathological settings like AD (Vercauteren et al., 2006). So beside the role of MAPK inhibitors in a potential tau phosphorylation and tau pathology as discussed both inhibitors may show benefit for an improvement of memory and mitochondrial impairment and thus play a neuroprotective role against Aβ-mediated deficits.

Another MAPK family inhibitor MW01-2-069A of the protein kinase p38α further profiled in Aβ-related studies and by molecular properties (Munoz et al., 2007). The selectivity in binding to the p38 isoform α could be reasoned with the favourable binding of the 4-pyridyl-residue in the ATP binding pocket which allows hydrogen bonding to the amide function of Gly110 of the protein backbone. Furthermore, the phenyl residue finds an optimized location in the neighbored hydrophobic binding pocket. P38α inhibitors have been discussed above to reduce the production of proinflammatory cytokines (Kim et al., 2004). MW01-2-069A was demonstrated to attenuate Aβ-mediated decreases of synaptophysin as a presynaptic protein. The downregulation of synaptophysin causes synaptic dysfunctions in the hippocampus and following hippocampal behavioral deficits. Favorably, the compound passes the blood brain barrier and proved to be metabolically stable to an extent of 70% (Munoz et al., 2007). However, the inhibitory activity in submicromolar ranges has to be considered critically with respect to later clinical studies.

The protein kinase Rock has been demonstrated to be activated in neurites surrounded by amyloid deposites (Petratos et al., 2008). The following outgrowth of the neurites could be protected by Rock inhibitor Y-27632. Moreover, an amyloid-induced loss of synapses could be retracted, so that novel synapse formation were observed. Furthermore the Rock inhibitor interfered with the APP processing by an increase of the α-secretase activity leading to less toxic Aβ (Quin et al., 2006).

1-Aza-9-oxafluorenes discovered as moderate cdk1 inhibitors first have been further developed as selective GSK3β inhibitors with a 3-carbonyl amide function at the 3-position of the molecular skeleton (Voigt et al., 2008) and a loss of the cdk1 inhibition. While the pyridine nitrogen atom of the 3-carbonyl derivatives showed optimized hydrogen bonding to the NH function of amino acid Val135 of the protein backbone, the NH amide function bound to carbonyl amide function of Thr138 via a water molecule. Further variations at the molecular scaffold led to the benzo-annelated lead compounds which further profiled as cdk1, gsk3β and cdk5 inhibitors (Tell et al., 2012a). The binding mode of those benzo-annelated naphtho compounds to the protein kinase backbone was demonstrated to be inverse compared to the early 1-aza-9-oxafluorenes with an orientation of the 3-substituent within the hydrophobic binding pocket nearby the gatekeeper amino acids (Voigt et al., 2008; Tell et al., 2012a). One nanomolar active 3-hydroxy compound showed exclusive cdk1, gsk3β and cdk5 inhibition properties and proved to be non-toxic in various cellular assays (Tell et al., 2012b). It effectively inhibited tau phosphorylation of various tau amino acids and has been suggested for further tau pathology studies in tau mice models.

## CHALLENGES

During the last decades of AD research it has become more obvious that AD is not only a multifactorial disease with various pathological events which contribute to the diseases progression but also a cross-linked disease. Aβ toxicity has been demonstrated to increase tau pathology. The linker has been GSK3β which itself is known to increase the production of Aβ. GSK3β has been known to be primed by cdk5 so that the inhibition of cdk5 reduces Aβ toxicity. Synaptic dysfunctions are not only mediated by a decrease of acetylcholine, but also by Aβ-induced increases of presynaptic proteins. Moreover, Aβ mediates inflammatory brain processes via protein kinases.

The low benefit of drugs which target one single target structure enforced studies for a potential multitargeting. The early strategy in this field concentrated on the combination of differently acting drugs by linkers like alkyl chains. The present outcome of these studies is poor. However, combining of two drugs by a linker leads to new molecules with changed molecular properties. Resulting enlarged molecular weights influence resorption processes and brain entry properties. It will be a challenge to develop a favourable combined drug for AD therapy.

Protein kinases have been identified as mainly disease influencing target structures. Their central role in tau pathology by tau phosphorylation is extended to disease-linking processes and makes them to most interesting target structures.

Limits in the development of inhibitors of such AD-relevant protein kinases have been pointed out: the brain entry of such small molecules, toxic cellular effects which accompany also recent inhibitors and highly active molecules. The latter aspect became obvious by recent studies with GSK3β inhibitors. None of the highly active inhibitors reached clinical trials. A micromolar inhibitor seems to be more promising.

What can be the conclusion from all these points? It will be of great interest to develop a drug which itself has such multitargeting properties. As protein kinases are promising target structures further developments shall concentrate on a multitargeting protein kinase inhibitor which has to be profiled in the various ways like brain entry abilities, low cellular toxicity and finally a sufficient but not a too strong activity just to regulate the protein kinases’ overactivities and not to interfere with their activities in normal cells. 1-Aza-9-oxafluorenes from a really perspective compound class on this way of a hopeful AD drug development.

## Conflict of Interest Statement

The authors declare that the research was conducted in the absence of any commercial or financial relationships that could be construed as a potential conflict of interest.

## References

[B1] Ashabi G. Ramin M. Azizi P. Taslimi Z. Alamdary S. Z. Haghparast A. (2012). ERK and p38 inhibitors attenuate memory deficits and increase CREB phosphorylation and PGC-1α levels in Aβ-injected rats. *Behav. Brain Res.* 232 165–173 10.1016/j.bbr.2012.04.006 22510382

[B2] Avila J. (2006). Tau phosphorylation and aggregation in Alzheimer’s disease pathology. *FEBS Lett.* 580 2922–2927 10.1016/j.febslet.2006.02.067 16529745

[B3] Baumann K. Mandelkov E. M. Biernat J. Piwnica-Worms H. Mandelkov E. (1993). Abnormal Alzheimer-like phosphorylation of tau protein by cyclin-dependent kinases cdk2 and cdk5. *FEBS Lett.* 336 417–424 10.1016/0014-5793(93)80849-P 8282104

[B4] Bennett B. L. Sasaki D. T. Murray B. W. O’Leary E. C. Sakata S. T. Xu W. (2001). SP-600125, an anthrapyrazolone inhibitor of c-Jun N-terminal kinase. *Proc. Nat. Acad. Sci. U.S.A.* 98 13681–13686 10.1073/pnas.251194298 PMC61101 11717429

[B5] Bettayeb K. Oumata N. Echalier A. Ferandin Y. Endicott J. A. Galons H. (2008). CR8, a potent and selective, roscovitine-derived inhibitor of cyclin-dependent kinases. *Oncogene* 27 5797–5807 10.1038/onc.2008.191 18574471

[B6] Birks J. (2006). Cholinesterase inhibitors for Alzheimer’s disease. *Cochrane Database Syst. Rev.* 2 CD005593 10.1002/14651858.CD005593 PMC9006343 16437532

[B7] Cruz J. C. Tseng H. C. Goldman J. A. Shih H. Tsai L. H. (2003). Aberrant Cdk5 activation by p25 triggers pathological events leading to neurodegeneration and neurofibrillary tangles. *Neuron* 40 471–483 10.1016/S0896-6273(03)00627-5 14642273

[B8] Demange L. Abdellah F. N. Lozach O. Ferandin Y. Gresh N. Meijer L. (2013). Potent inhibitors of CDK5 derived from roscovitine: synthesis, biological evaluation and molecular modelling. *Bioorg. Med. Chem. Lett.* 23 125–131 10.1016/j.bmcl.2012.10.141 23218601

[B9] Flajolet M. He G. Heiman M. Lin A. Nairn A. C. Greengard P. (2007). Regulation of Alzheimer’s disease amyloid-beta formation by casein kinase 1. *Proc. Natl. Acad. Sci. U.S.A.* 104 4159–4164 10.1073/pnas.0611236104 17360493 PMC1820725

[B10] Hamdane M. Bretteville A. Sambo A. V. Schindowski K. Begard S. Delacourte A. (2005). P25/cdk5-mediated retinoblastoma phosphorylation is an early event in neuronal cell death. *J. Cell Sci.* 118 1291–1298 10.1242/jcs.01724 15741232

[B11] Hampel H. Ewers M. Burger K. Annas P. Mörtberg A. Bogstedt A. (2009). Lithium trial in Alzheimer’s disease: a randomized, single-blind, placebo-controlled, multicenter 10-week study. *J. Clin. Psychiatry* 70 922–931 10.4088/JCP.08m04606 19573486

[B12] Hollander B. A. Bennett G. S. Shaw G. (1996) Localisation of sites in the tail-domain of the middle molecular mass neurofilament subunit phosphorylated by a neurofilament-associated kinase and by casein kinase I. *J. Neurochem.* 66 412–420 10.1046/j.1471-4159.1996.66010412.x 8522982

[B13] Hoshi M. Takashima A. Noguchi K. Murayama M. Sato M. Kondo S. (1996). Regulation of mitochondrial pyruvate dehydrogenase activity by tau protein kinase 1/glycogen synthase kinase 3beta in brain. *Proc. Natl. Acad. Sci. U.S.A.* 93 2719–2723 10.1073/pnas.93.7.2719 8610107 PMC39697

[B14] Iqbal K. Grundke-Iqbal I. (2002). Alzheimer disease is multifactorial and heterogeneous. *Neurobiol. Aging* 21 901–902 10.1016/S0197-4580(00)00191-3 11124439

[B15] Iqbal K. Grundke-Iqbal I. (2008). Alzheimer neurofibrillary degeneration: significance, etiopathogenesis, therapeutics and prevention. *J. Cell. Mol. Med.* 12 38–55 10.1111/j.1582-4934.2008.00225.x 18194444 PMC3139457

[B16] Kim S. H. Smith C. J Van Eldik L. J. (2004). Importance of MAPK pathways for microglial proinflammatory cytokine IL-1 beta production. *Neurobiol. Aging* 25 431–439 10.1016/S0197-4580(03)00126-X 15013563

[B17] Kimura R. Kamino K. Yamamoto M. Nuripa A. Kida T. Kazui H. (2007). The DYRK1A gene, encoded in chromosome 21 down syndrome critical region, bridges between beta-amyloid production and tau phosphorylation in Alzheimer disease. *Hum. Mol. Genet.* 16 15–23 10.1093/hmg/ddl437 17135279

[B18] Knippschild U. Gocht A. Wolff S. Huber N. Lohler J Stöter M. (2005). The casein kinase 1 family: participation in multiple cellular processes in eukaryotes. *Cell. Signal.* 17 675–689 10.1016/j.cellsig.2004.12.011 15722192

[B19] Krug M. Hilgeroth A. (2008). Recent advances in the development of multi-kinase inhibitors. *Mini Rev. Med. Chem.* 8 1312–1327 10.2174/138955708786369591 18991750

[B20] Kudo K. Wati H. Qiao C. Arita J. Kanba S. (2005). Age-related disturbance of memory and CREB phosphorylation in CA1 area of hippocampus of rats. *Brain Res.* 1054 30–37 10.1016/j.brainres.2005.06.045 16054117

[B21] Kuret J. Johnson G. S. Cha D. Christensen E. R. Demaggio A. J. Hoekstra M. F. (1997). Casein kinase 1 is tightly associated with paired-helical filaments isolated from Alzheimer’s diseases brain. *J. Neurochem.* 69 2506–2515 10.1046/j.1471-4159.1997.69062506.x 9375684

[B22] Leroy K. Yilmaz Z. Brion J. P. (2007). Increased level of GSK-3beat in Alzheimer’s disease and accumulation in agyrophilic grains and in neurons at different stage of neurofibrillary degeneration. *Neuropathol. Appl. Neurobiol.* 33 43–55 10.1111/j.1365-2990.2006.00795.x 17239007

[B23] Liao Y. C. Andrews R. C. (2007). Targeting protein multiple conformations: a structure-based strategy for kinase drug design. *Curr. Top. Med. Chem.* 7 1394–1407 10.2174/156802607781696783 17692028

[B24] Loidreau Y. Marchand P. Dubouilh-Benard C. Nourrisson M.-R. Duflos M Loaëc N. (2013). Synthesis and biological evaluation of N-aryl 7-methoxybenzo[b]furo[3,2-d]pyrimidin-4-amines and their *N*-arylbenzo[b]thieno[3,2-d]pyrimidin-4-amine analogues as dual inhibitors of CLK1 and DYRK1A kinases. *Eur. J. Med. Chem.* 59 283–295 10.1016/j.ejmech.2012.11.030 23237976

[B25] Luna-Medina R. Cortes-Canteli M. Sanchez-Galiano S. Morales-Garcia J. A. Martinez A. Santos A. (2007). NP031112, a thiadiazolidinone compound, prevents inflammation and neurodegeneration under excitotoxic conditions: potential therapeutic role in brain disorders. *J. Neurosci.* 27 5766–5776 10.1523/JNEUROSCI.1004-07.2007 17522320 PMC6672766

[B26] Marco A. M. Karla I. L. Victoria C. Martha A. D. Raul M. (2010). Tau oligomers and aggregation in Alzheimer’s disease. *Neurochemistry* 112 1353–1367 10.1111/j.1471-4159.2009.06511.x 19943854

[B27] Martin L. Latypova X. Terro F. (2011). Post-translational modifications of tau protein: implications for Alzheimer’s disease. *Neurochem. Int.* 58 458–471 10.1016/j.neuint.2010.12.023 21215781

[B28] Martin L. Latypova X. Wilson C. M. Magnaudeix A. Perrin M.-L. Yardin C. (2013). Tau protein kinases: involvement in Alzheimer’s disease. *Ageing Res. Rev.* 12 289–309 10.1016/j.arr.2012.06.003 22742992

[B29] Mashhoon N. DeMaggio A. J. Tereshko V. Bergmeier S. C. Egli M. Hoekstra M. F. (2000). Crystal structure of a conformation-selective casein kinase-1 inhibitor. *J. Biol. Chem.* 275 20052–20060 10.1074/jbc.M001713200 10749871

[B30] Mattson M. P. (2004). Secreted forms of beta-amyloid precursor protein modulate dendrite outgrowth and calcium responses to glutamate in cultured embryonic hippocampal neurons. *J. Neurobiol.* 25 439–450 10.1002/neu.480250409 7915758

[B31] McShane R. Aerosa S. Minakaran N. (2006). Memantine for dementia. *Cochrane Database. Syst. Rev.* 2 CD003154 10.1002/14651858.CD003154.pub5 16625572

[B32] Meijer L. Raymond E. (2003). Roscovitine and other purines as kinase inhibitors. From starfish oocytes to clinical trials. *Acc. Chem. Res.* 36 417–425 10.1021/ar0201198 12809528

[B33] Mohandas E. Rajmohan V. Raghunath B. (2009). Neurobiology of Alzheimer’s disease. *Indian J. Psychiatry* 51 55–61 10.4103/0019-5545.44908 19742193 PMC2738403

[B34] Munoz L. Ranaivo H. R. Saktimayee M. R. Wenhui H. Craft J. M. McNamara L. K. (2007). A novel p38α MAPK inhibitor suppresses brain proinflammatory cytokine up regulation and attenuates synaptic dysfunction and behavioural deficits in an Alzheimer’s disease mouse model. *J. Neuroinflammation* 4 21–34 10.1186/1742-2094-4-21 17784957 PMC2014744

[B35] Noble W. Olm V. Takata K. Casey E. Mary O. Meyerson J. (2003). Cdk5 is a key factor in tau aggregation and tangle formation in vivo. *Neuron* 38 555–565 10.1016/S0896-6273(03)00259-9 12765608

[B36] Ohori M. Kinoshita T. Okubo M. Sato K. Yamazaki A. Arakawa H. (2005). Identification of a selective ERK inhibitor and structural determination of the inhibitor-ERK2 complex. *Biochem. Biophys. Res. Commun.* 336 357–363 10.1016/j.bbrc.2005.08.082 16139248

[B37] Pagano M. A. Poletto G. DiMaira G. Cozza G. Ruzzane M. Sarno S. (2007). Tetrabromocinnamic acid (TBCA) and related compounds represent a new class of specific protein kinase CK2 inhibitors. *ChemBioChem* 8 129–139 10.1002/cbic.200600293 17133643

[B38] Pakaski M. Kalman J. (2008). Interactions between the amyloid and cholinergic mechanisms in Alzheimer’s disease. *Neurochem. Int.* 53 103–111 10.1016/j.neuint.2008.06.005 18602955

[B39] Pardridge W. M. (2005). The blood-brain barrier: bottleneck in brain drug development. *NeuroRx* 2 3–14 10.1602/neurorx.2.1.3 15717053 PMC539316

[B40] Patrick G. N. Zukerberg L. Nikolic M. de la Monte S. Dikkes P. Tsai L. H. (1999). Conversion of p35 to p25 deregulates Cdk5 activity and promotes neurodegeneration. *Nature* 402 615–622 10.1038/45159 10604467

[B41] Pei J. J. Braak H. An W. L. Winblad B. Cowburn R. F. Iqbal K. (2002). Up-regulation of mitogen-activated protein kinases ERK1/2 and MEK1/2 is associated with the progression of neurofibrillary degeneration in Alzheimer’s disease. *Brain Res.* 109 45–55 10.1016/S0169-328X(02)00488-6 12531514

[B42] Pei J. J. Sjogren M. Winblad B. (2008). Neurofibrillary degeneration in Alzheimer’s disease: form molecular mechanisms to identification of drug targets. *Curr. Opin. Psychiatry* 21 555–561 10.1097/YCO.0b013e328314b78b 18852562

[B43] Pei J. J. Tanaka T. Tung Y. C. Braak E. Iqbal K. Grundke-Iqbal I. (1997). Distribution levels, and activity of glycogen synthase kinase-3 in Alzheimer disease brain. *J. Neuropathol. Exp. Neurol.* 56 70–78 10.1097/00005072-199701000-00007 8990130

[B44] Petratos S. Li Q. X. Gorge A. J. Hou X. Kerr M. L. Unabia S. E. (2008). The beta- amyloid protein of Alzheimer’s disease increases neuronal CRMP-2 phosphorylation by a Rho-GTP mechanism. *Brain* 131 90–108 10.1093/brain/awm260 18000012

[B45] Philpott K L. Facci L. (2008). MAP kinase pathways in neuronal cell death. *CNS Neurol. Disord. Drug Targets* 7 83–97 10.2174/187152708783885129 18289035

[B46] Piau A. Nourhasémi F. Hein C. Caillaud C. Vellas B. (2011). Progress in the development of new drugs in Alzheimer’s disease. *J. Nutr. Health Aging* 15 45–57 10.1007/s12603-011-0012-x 21267520 PMC12879621

[B47] Quin W. Yang T. Ho L. Zhao Z. Wang J. Chen L. (2006). Neuronal SIRT1 activation as a novel mechanism underlying the prevention of Alzheimer disease amyloid neuropathology by calorie restriction. *J. Biol. Chem.* 281 21745–21754 10.1074/jbc.M602909200 16751189

[B48] Raina P. Santaguida P. Isamaila A. Patterson C. Cowan D. Levine M. (2008). Effectiveness of cholinesterase inhibitors and memantine for treating dementia: evidence review for a clinical practice guideline. *Ann. Intern. Med.* 148 379–397 10.7326/0003-4819-148-5-200803040-00009 18316756

[B49] Rojo L. E. Fernandez J. A. Maccioni A. A. Jimenez J. M. Maccioni R. B. (2008). Neuroinflammation: implications for the pathogenesis and molecular diagnosis of Alzheimer’s disease. *Arch. Med. Res.* 39 1–16 10.1016/j.arcmed.2007.10.001 18067990

[B50] Schwab C. DeMaggio A. J. Ghoshal N. Binder L. I. Kuret J McGeer P. L. (2000). Casein kinase 1 delta is associated withpathological accumulation of tau in several neurodegenerative diseases. *Neurobiol. Aging* 21 503–510 10.1016/S0197-4580(00)00110-X 10924763

[B51] Seifert A. Allan L. A. Clarke P. R. (2008). DYRK1A phosphorylates caspase 9 at an inhibitory site and is potently inhibited in human cells by harmine. *FEBS J.* 275 6268–6280 10.1111/j.1742-4658.2008.06751.x 19016842

[B52] Sengupta A. Kabat J. Novak M. Wu Q. Grungke-Iqbal I. Iqbal K. (1998). Phosporylation of tau at both Thr213 and Ser262 is required for maximal inhibition of its binding to microtubules. *Arch. Biochem. Biophys.* 357 299–309 10.1006/abbi.1998.0813 9735171

[B53] Shaftel S. S. Griffin W. S O’Banion M. K. (2008). The role of interleukin-1 in neuroinflammation and Alzheimer disease: an evolving perspective. *J. Neuroinflammation* 5 7 10.1186/1742-2094-5-7 PMC2335091 18302763

[B54] Shen C. Chen Y. Liu H. Zhang K. Zhang T. Lin A. (2008). Hydrogen peroxide promotes Abeta production through JNK-dependent activation of gamma-secretase. *J. Biol. Chem.* 283 17721–17730 10.1074/jbc.M800013200 18436531 PMC2427353

[B55] Takashima A. Noguchi K. Michel G. Mercken M. Hoschi M. Ishiguro K. (1996). Exposure of rat hippocampal neurons to amyloid beta peptide (25–35) induces the inactivation of phosphatidyl inositol-3-kinase and the activation of tau protein kinase 1/glycogen synthase kinase-3-beta. *Neurosci. Lett.* 203 33–36 10.1016/0304-3940(95)12257-5 8742040

[B56] Tariot P. N. Aisen P. S. (2009). Can lithium or valproate untie tangles in Alzheimer’s disease? *J. Clin. Psychiatry* 70 919–921 10.4088/JCP.09com05331 19573485

[B57] Tell V. Mahmoud K. A. Wichapong K. Schächtele C. Totzke F. (2012a). Novel aspects in structure activity relationships of profiled 1-aza-9-oxafluorenes as inhibitors of Alzheimer’s diseases-relevant kinases cdk1, cdk5 and gsk3β. *Med. Chem. Commun.* 3 1413–1418 10.1039/C2MD20201H

[B58] Tell V. Holzer M. Herrmann L. Mahmoud K. A. Schächtele C. Totzke F. (2012b). Multitargeted drug development: discovery and profiling of dihydroxy substituted 1-aza-9-oxafluorenes as lead compounds targeting Alzheimer disease relevant kinases. *Bioorg. Med. Chem. Lett.* 22 6914–6918 10.1016/j.bmcl.2012.09.006. Epub 2012 Sep 13. 23039927

[B59] Terry A. V. Jr. Buccafusco J. J. (2003). The cholinergic hypothesis of age and Alzheimer’s disease-related cognitive deficits: recent challenges and their implications for novel drug development. *J. Pharmacol. Exp. Ther.* 306 821–827 10.1124/jpet.102.041616 12805474

[B60] Tian Q. Wang J. (2002). Role of serine/threonine protein phosphatase in Alzheimer’s disease. *Neurosignals* 11 262–269 10.1159/000067425 12566927

[B61] Underwood D. C. Osborn R. R. Kotzer C. J. Adams J. L. Lee J. C. Webb E. F. (2000). SB-23063, a potent p38 MAP kinase inhibitor, reduces inflammatory cytokine production, airway eosinophil infiltration, and persistence. *J. Pharmacol. Exp. Ther.* 293 281–288 10734180

[B62] U.S. National Institutes of Health. (2010). Efficacy study of oral seliciclib to treat non-small cell lung cancer. *Cyclacel Pharmaceuticals, Inc.* Available at: http://clinicaltrials.gov/ct2/show/NCT00372073?/term=Selciclib&rank=2 (accessed on December 06, 2010).

[B63] Utreras E. Maccioni R. Gonzalez-Billaut C. (2009). Cyclin-dependent kinase 5 activator p35 overexpression and amyloid beta synergism increase apoptosis in cultured neuronal cells. *Neuroscience* 161 978–987 10.1016/j.neuroscience.2009.04.002 19362124

[B64] Vercauteren K. Pasko R. A. Gleyzer N. Marino V. M. Scarpulla R. C. (2006). PGC-1-related coactivator: immediate early expression and characterization of a CREB/NRF-1 binding domain associated with cytochrome *c* promoter occupancy and respiratory growth. *Mol. Cell Biol.* 26 7409–7419 16908542 10.1128/MCB.00585-06 PMC1636882

[B65] Voigt B. Krug M. Schächtele C. Totzke F. Hilgeroth A. (2008). Probing Novel 1-Aza-9-oxafluorenes as selective GSK-3β inhibitors. *ChemMedChem* 3 120–126 10.1002/cmdc.200700175 18000938

[B66] Wen Y. Planel E. Herman M. Figueroa H. Y. Wang L. Liu L. (2008). Interplay between cyclin-dependent kinase 5 and glycogen synthase kinase 3 beta mediated by neuregulin signalling leads to differential effects on tau phosphorylation and amyloid precursor protein processing. *J. Neurosci.* 28 2624–2632 10.1523/JNEUROSCI.5245-07.2008 18322105 PMC6671193

[B67] Yang E. J. Ahn Y. S. Chung K. C. (2001). Protein kinase Dyrk1 activates cAMP response element-binding protein during neuronal differentiation in hippocampal progenitor cells. *J. Biol. Chem.* 276 39819–39824 10.1074/jbc.M104091200 11518709

[B68] Yasojima K. Kurte J. De Maggio A. J. McGeer E. McGeer P. L. (2000). Casein kinase 1 delta mRNA is upregulated in Alzheimer disease brain. *Brain Res.* 865 116–120 10.1016/S0006-8993(00)02200-9 10814741

[B69] Zhang J. Yang P. L. Gray N. S. (2009). Targeting cancer with small molecule kinase inhibitors. *Nat. Rev. Cancer* 9 28–29 10.1038/nrc2559 19104514 PMC12406740

[B70] Zhu X. Rottkamp C. A. Boux H. Takeda A. Perry G. Smith M. A. (2000). Activation of p38 kinase links tau phosphorylation, oxidative stress, and cell cycle-related events in Alzheimer’s disease. *J. Neuropathol. Exp. Neurol.* 59 880–888 10.1046/j.1471-4159.2001.00046.x 11079778

